# Freeze for action: neurobiological mechanisms in animal and human freezing

**DOI:** 10.1098/rstb.2016.0206

**Published:** 2017-02-27

**Authors:** Karin Roelofs

**Affiliations:** Donders Institute for Brain Cognition and Behaviour and Behavioural Science Institute, Radboud University Nijmegen, Kapittelweg 29, 6525 EN, Nijmegen, The Netherlands

**Keywords:** freezing, defence cascade, parasympathetic and sympathetic autonomic nervous system, freeze-fight-flight, neural fear and defence circuits

## Abstract

Upon increasing levels of threat, animals activate qualitatively different defensive modes, including freezing and active fight-or-flight reactions. Whereas freezing is a form of behavioural inhibition accompanied by parasympathetically dominated heart rate deceleration, fight-or-flight reactions are associated with sympathetically driven heart rate acceleration. Despite the potential relevance of freezing for human stress-coping, its phenomenology and neurobiological underpinnings remain largely unexplored in humans. Studies in rodents have shown that freezing depends on amygdala projections to the brainstem (periaqueductal grey). Recent neuroimaging studies in humans have indicated that similar brain regions may be involved in human freezing. In addition, flexibly shifting between freezing and active defensive modes is critical for adequate stress-coping and relies on fronto-amygdala connections. This review paper presents a model detailing these neural mechanisms involved in freezing and the shift to fight-or-flight action. Freezing is not a passive state but rather a parasympathetic brake on the motor system, relevant to perception and action preparation. Study of these defensive responses in humans may advance insights into human stress-related psychopathologies characterized by rigidity in behavioural stress reactions. The paper therefore concludes with a research agenda to stimulate translational animal–human research in this emerging field of human defensive stress responses.

This article is part of the themed issue ‘Movement suppression: brain mechanisms for stopping and stillness’.

## 1. Introduction

Imagine you are standing in your office and all of a sudden a man walks in and attacks you with a knife. What would you do? In 2007, a Dutch police officer became seriously wounded in such an armed attack in a police office in Amsterdam. Upon the attack, she froze for a moment and then decided to shoot. The offender died on the spot. Later analyses of this shocking event made people realize that if the officer had frozen slightly longer, more people might have been injured. On the other hand, if she had decided to shoot immediately after detecting the armed man, she might not have been wounded so badly.

Police officers are trained to deal with acute threat and to inhibit their automatic action tendencies in order to optimize adequate response capacity. In stressful situations, however, most people tend to fall back on primary ‘freeze–fight–flight’ tendencies and have great difficulty controlling their actions or shifting flexibly between passive freezing and active fight-or-flight. Insight into how these defensive reactions are controlled in the brain is relevant for individuals in high-risk professions who have to perform optimally under stress. In addition, it may be important for improving remedies for psychopathologies that are characterized by a rigidity in defensive stress reactions. For example, anxiety has been associated with persistent freeze and flight tendencies [1,2], whereas aggression is related to reduced freezing and heightened fight tendencies [3–5].

This article reviews recent insights into the phenomenology of threat-induced freezing in humans and animals. Particularly, it focuses on how we can control automatic defensive threat reactions. What neural mechanisms support flexible shifting between passive ‘freezing’ and active ‘fight-or-flight’ modes? Do we see individual differences in these automatic action tendencies and might it be possible to influence or ‘train’ them? Before addressing these questions, I first describe the phenomenology of freezing and fight-or-flight reactions as well as the psychophysiological and neural mechanisms associated with these threat-related defensive states.

## 2. Phenomenology

The coevolution of prey and predator has evolved into qualitatively different defensive action repertoires that animals display when facing predator threat [6–8]. Freezing is activated at intermediate levels of predator threat. It is a state of attentive immobility serving to avoid detection by predators and to enhance perception [9,10]. Besides immobility, an important feature of freezing is the parasympathetically induced heart rate deceleration, also called ‘bradycardia’. Freezing differentiates with the sympathetically dominated fight-or-flight response activated during imminent predation threat [8]. Especially, upon threat, both sympathetic and parasympathetic branches of the autonomic nervous system are simultaneously activated and only in case of parasympathetic dominance do we observe defensive freezing.

Freezing was originally referred to as crouching [11], a complete absence of movement except for movements associated with respiration and tense body posture that result from increased muscle tone in this defensive state [8,12]. Later, well-controlled animal studies consistently observed bradycardia associated with freezing [13,14]. Other features, such as reduced vocalizations and changes in body temperature have been described as well but have not been observed as consistently [15] (see box 1 for an overview of additional phenomena).

Box 1. **Freezing, a state of parasympathetic dominance.**

When a stimulus or a situation is perceived to be threatening, the brain activates many neuronal circuits to adapt to the demand, the most well-known being the autonomic nervous system (ANS). During freezing, the two counteracting branches of the ANS, the sympathetic and parasympathetic nervous systems, become activated [16]. It is important to realize that physiological parameters of freezing therefore consist of both sympathetic and parasympathetic features, which vary depending on which system is dominant at a certain point in time. *Sympathetic* nervous system activity is expressed by increased arousal and physical symptoms that support the freezing response: increased heart rate and cardiac output, increased arterial pressure, inhibition of digestive function and increased respiration, in its turn increasing perfusion of active tissue. There is also increased muscle tone and pain suppression [14,17].

Activation of the *parasympathetic* branch of the ANS during freezing causes heart rate deceleration [14,18]. Parasympathetic dominance during freezing has therefore been associated with a net heart rate deceleration or a reduced heart rate acceleration [19]. Freezing can also be associated with altered respiration rates and vocalizations. Respiration during freezing in rats is rapid until they start to vocalize ultrasonically. At that moment the respiratory rate drops because ultrasonic vocalizations require long periods of expiration. Whereas reduced vocalization in rats has been associated with fear during acute threat, increases in vocalization have been observed during freezing reactions to anxiety associated with potential threat [20,21].

Freezing is a universal fear response, observed both in reaction to conditioned (learned) or unconditioned (acutely threatening) stimuli or situations [22]. It is manifested as part of a repertoire of species-specific defensive responses, with some species showing a strong innate preference for freezing and other species hardly ever reacting by freezing [23]. State as well as trait factors and environmental ones play a role in the shaping of defensive behaviour. As far as environmental factors are concerned, the distance from the predator and the presence of escape routes play an important role in determining whether species freeze or not. Distal threat evokes longer freezing reactions than proximal threat. With escape routes available, freezing is shorter and more likely followed by fleeing compared with a situation were no escape routes are available [24]. Examples of state factors are age and incubation. For instance, young rats may show freezing but do not yet show the typical reduction of heart rate acceleration [25] and incubating hens typically show bradycardia, whereas non-incubating hens show tachycardia [26]. An example of an important trait factor influencing the manifestation of defensive reactions is anxiety. It has been well documented that rats with a genetic predisposition to anxiety show more freezing than non-anxious ones [27].

**Figure 1.**
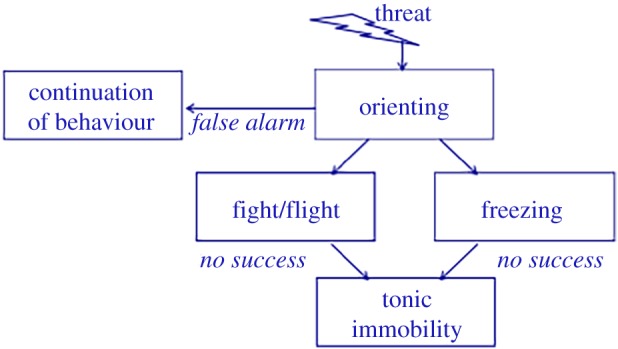
Schematic of routes of defensive behaviour (adapted from Hagenaars *et al.* [15]). (Online version in colour.)

Freezing should be differentiated from other threat-induced states characterized by immobility, such as orienting and tonic immobility (figure 1). During orienting, attention is directed to a novel stimulus or situation. This state is often accompanied by reduced motion. Orienting and freezing both share an attentive immobility and are therefore difficult to tell apart. Although freezing is sometimes regarded as part of the orienting response occurring immediately upon threat detection [28], there are important differences between orienting and freezing. First, orienting is subject to habituation and freezing is not [29]. Second, systematic investigation of threat-induced freezing in active action-preparation paradigms has indicated that the heart rate deceleration accompanied by immobility tends to follow a pattern of response preparation, becoming stronger as a function of action preparation [5]. Orienting is also typically observed when a predator is slowly approaching its prey and shows intermittent episodes of attentive immobility to reorient from its newly reached position. Tonic immobility is yet another defensive state that should be distinguished from freezing. Tonic immobility, like freezing, is featured by the absence of movement in response to severe threat. Importantly however, while freezing can take place early in the defence cascade, tonic immobility occurs later, for example, in case of circa-strike physical contact, when fight, flight and freezing are no longer optimal for survival [7,30]. In contrast to freezing, physical features of tonic immobility remain largely unclear and are often contradictory. Some studies report heart rate increases [31] and others describe heart rate decreases [32]. Particularly, hypotension and unresponsiveness [33–35] differentiate tonic immobility from freezing. In contrast to passive tonic immobility (‘playing dead’), freezing actively prepares the animal for further defensive responses [24,36], as suggested by increased rather than decreased startle responses during freezing [37,38].

## 3. Neural mechanisms of freezing

**Figure 2.**
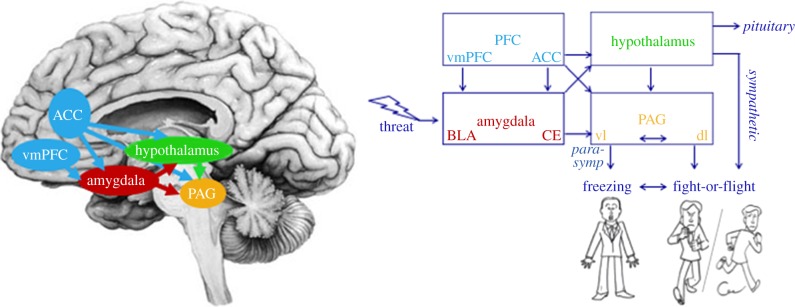
Schematic of brain structures involved in the control of freezing and fight-or-flight reactions to threat. When threat is processed in the basolateral (BLA) parts of the amygdala, direct connections from the central nucleus of the amygdala (CE) to the ventrolateral periaqueductal grey (vlPAG) mediate freezing by (i) activating the vagal pathway, which in turn regulates parasympathetic (parasymp) heart rate deceleration and (ii) by regulating muscular activity by at least two routes: vlPAG activation (i) inhibits activation of fight-or-flight responses by the dorsolateral (dl)PAG and (ii) modulates premotor neurons projecting to the spinal cord via the rostral ventral medulla. Preservation of muscle tone during freezing is enabled by projections to the lateral hypothalamus. This area also controls sympathetic visceral reactions and activates the pituitary as part of the hypothalamus–pituitary–adrenal (HPA) axis. Shifting between passive and active defensive modes is implemented by the ventromedial prefrontal cortex (vmPFC) and, in particular, the anterior cingulate cortex (ACC), which in turn projects to the CE of the amygdala and to the vlPAG.

The amygdala plays a key role in defensive behaviour and switching between defensive modes in rodents [39–42]. Stimulation of the central nucleus of the amygdala results in freezing, bradycardia and pupil dilation [43], whereas lesions of the same area block both autonomic and behavioural fear reactions [40,44]. Amygdala projections to the lateral hypothalamus mediate autonomic sympathetic responses (box 1), whereas projections to the medullar nuclei control parasympathetic effects through efferent vagal fibres that originate from the nucleus ambiguous [45,46]. However, connections from the central nucleus of the amygdala (CE) to the periaqueductal grey (PAG) [47] are responsible for behavioural aspects of the defence cascade. The PAG is a midbrain region implicated in several homoeostatic processes including fear, pain and analgesia [48,49]. In particular, the ventrolateral (vl) PAG is implicated in the freezing response. Lesions of the vlPAG [14,50,51] but not of the dorsal or dorsolateral (dl) PAG [52] disrupt freezing. See figure 2 for a schematic overview of brain regions contributing to freezing and fight-or-flight reactions. dlPAG activation is predominantly associated with active defensive behaviours such as fight-or-flight [53]. Stimulation of the (rodent) dlPAG indeed resulted in sudden activity bursts associated with panic behaviour and non-directional fight-or-flight [54]. There are, however, instances where dlPAG activation can result in freezing behaviour in addition to the active defensive reactions. In particular, when the animal is exposed to immediate unconditioned threat cues, dlPAG activation can—helped by afferent projections from the hypothalamus and superior colliculus—result in quiescent behaviour [55]. Therefore, it has been suggested that although vlPAG is predominantly involved in freezing to aversive conditioned stimuli, dlPAG is predominantly involved in unconditioned defensive responses [55,56].

## 4. The ventrolateral periaqueductal grey as a brake on the system

During the defensive state of freezing, the vlPAG serves as a brake on threat-related arousal systems and puts the fight-or-flight reactions on hold [14,57]. When vlPAG activation is blocked, rats show fear-related physiological responses (increased blood pressure and muscle tone) but no immobility or bradycardia [14]. In contrast, electrical or chemical stimulation of the vlPAG produces freezing [58,59]. The idea is that vlPAG inhibits the phasic but not the tonic component of motor function, resulting in a stage where the animal is aroused and has increased muscle tone but shows inhibition of the motor response. The animal is optimally prepared for action after the brake has been released. This is exactly what differentiates the state of freezing from a resting state or learned helplessness-related immobility. The most likely pathway via which vlPAG inhibits motor activity is via the rostral ventral medulla, activating premotor neurons that project to the spinal cord [14]. In addition, the vlPAG blocks fight-or-flight reactions generated by the lateral and dlPAG and activates the vagal pathway via the dorsal motor nucleus, which in turn regulates parasympathetic heart rate decelerations that are the autonomic equivalent of the freezing response [14]. Opioid-mediated analgesia responses during freezing are mediated by the vlPAG projections to the rostral ventromedial medulla projecting to opioid receptors in the spinal cord [60,61]. Thus, vlPAG is not only implicated in tonic immobility but also in heart rate deceleration and analgesia during freezing. Projections of the ventromedial prefrontal cortex (vmPFC) and the perigenual anterior cingulate cortex (ACC) in particular to the CE of the amygdala in turn facilitate rapid shifting between passive and active defence modes [41,62].

## 5. The role of stress hormones

During stress exposure, rapid activation of the sympathomedullary system (SAM) results in the release of the neurotransmitters adrenaline (epinephrine) and noradrenaline (norepinephrine). The sympathetic branch of the autonomic nervous system and associated reactions (involving pupil dilation, heart rate increase, increased muscle tone and rapid onset of fight-or-flight and freezing reactions) is largely driven by (nor) adrenaline, including noradrenergic projections from the locus coerulus to the dlPAG [55,63,64]. The parasympathetic branch of the autonomic nervous system and associated freezing reactions are largely driven by the neurotransmitter acetylcholine [65], as is the switch between freezing and active fear responses [41]. Activation of the hypothalamus–pituitary–adrenal (HPA) axis in turn results in the release of corticotrophin-releasing hormone (CRH), adrenocorticotropin hormone (ACTH) and cortisone (or cortisol in humans). CRH is essential for coordinating behavioural and metabolic threat reactions in the amygdala and many other brain regions, and facilitates expression of freezing in primates and rodents [12,66,67]. Basal and stress-induced cortisol levels have also been associated with increased freezing in primates and rodents, respectively [68,69]. Glucocorticoids play an important role in the normal development of defensive freezing. Preventing cortisone release in newborn rats by removing the adrenals, leads to impaired freezing, which can be restored by cortisol administration [21]. On the other hand, maternal care and postnatal handling of rats reduce cortisol stress responses later in life and have been associated with reduced freezing responses [70]. Interestingly, a positive relation between endogenous cortisol levels, on the one hand, and freezing and fear bradycardia, on the other, has also been found in human infants, whereas there was no such relation in the case of more sympathetically driven fear behaviours [71].

There are many other hormones and peptides known to affect freezing, including progesterone, testosterone, oestrogen, oxytocin and vasopressin [41,72]. Oxytocin may, for example, affect the shift from freezing to active defensive responses by acting on cholinergic transmission in the lateral CE of the amygdala and the ACC, but also by inhibiting vasopressin neurons in the medial CE that project to the vlPAG [41]. These hormones and peptides also act on other neurotransmitter systems implicated in the expression of freezing, including gamma-aminobutyric acid (GABA) dopamine and serotonin. GABA tonically inhibits defensive behaviour in the amygdala, hypothalamus and the PAG, an effect opposed by excitatory amino acids [41]. Serotonin release in the dlPAG and in the rostral ventrolateral medulla inhibits active fight-or-flight behaviours [73]. Interestingly, there are indications that endogenous serotonin in these regions originates not only from the dorsal raphe nucleus but also from the vlPAG, suggesting an additional mechanism by which vlPAG activity can inhibit dlPAG-driven fight-or-flight reactions [73].

## 6. Freezing in humans

**Figure 3.**
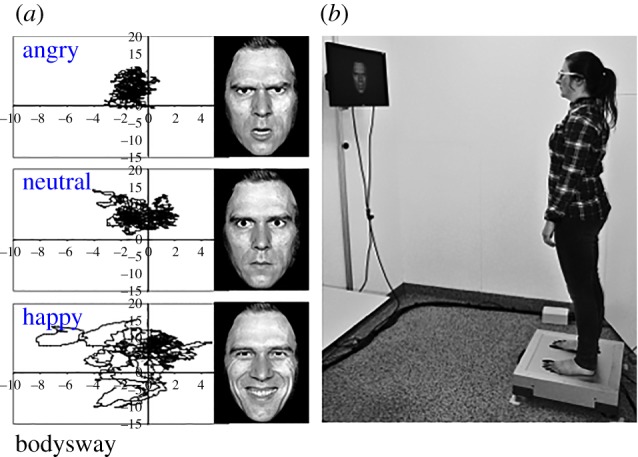
(*b*) Stabilometric force platform registering body sway in terms of displacements in the centre of pressure during picture-viewing. (*a*) Example of time series of body sway displacements (in millimetres in the anterior–posterior as well as lateral dimensions) in response to angry, neutral and happy faces (adapted from Roelofs *et al*. [1]).

Research on threat-induced freezing reactions in humans has largely focused on induction of fear bradycardia by aversive picture-viewing and by threat of shock. A well-established paradigm involves exposure to pictorial stimuli taken from the International Affective Picture System (IAPS) [74]. Studies using this paradigm have shown that autonomic responses to affective stimuli, which vary on dimensions of valence and arousal, closely resemble autonomic responses associated with defensive behaviours in rodents [9,75]. For instance, negatively valenced and highly arousing pictures elicit sympathetic changes such as galvanic skin responses [9] and pupil dilation [76]. Interestingly, numerous studies have demonstrated that such stimuli can also induce heart rate deceleration or bradycardic [9,76,77] that may be associated with sustained attentional processing of the stimuli [78]. More recently, studies have attempted to associate this heart rate response directly to bodily freezing behaviour in humans. Posturographical analyses, using a stabilometric force platform, have confirmed that bradycardic responses are accompanied by reduced locomotion as measured by postural sway on a stabilometric force platform ([1,2,79,80]; figure 3). Similar reductions in heart rate can be induced by threat of a mild electric shock [5].

### (a) Freezing and perceptual sensitivity

Using threat of shock paradigms, a few interesting observations have been made in humans, namely that freezing is related to action preparation and is associated with altered perceptual sensitivity. First, in a visual discrimination task, Lojowska *et al*. [11] observed that fear bradycardia induced by threat of shock was associated with improved detection of low-spatial frequency (LSF) cues, at the expense of high spatial frequency detection. These findings indicated that freezing is associated with better detection of coarse rather than detailed visual information and may follow the so-called better safe than sorry principle (see box 2 for more details).

Box 2. **Freezing facilitates visual perception of low spatial frequency information.**

To test effects of threat-induced freezing on perceptual sensitivity for low versus high spatial frequency information in humans, Lojowska *et al*. [10] used a visual discrimination task in threat and safe conditions. In the first part of the experiment, a fear-conditioning procedure was used to couple a visual cue (CS+; threat condition) to a mild but aversive electric shock and another cue to the absence of shock (CS−; safe condition). Panel *a* illustrates relative heart rate deceleration (fear bradycardia) in the threat (versus safe) condition, indexing parasympathetic-dominated freezing. After a variable time interval after cue presentation, a target appeared consisting of a tilted Gabor patch representing low (LSF) or high spatial frequency (HSF) information. In a visual discrimination, paradigm subjects had to indicate whether the target was tilted to the left or to the right with respect to the upright position. Tilt of the Gabor patches was adapted to set the performance on 75% correct. Under threat of shock, performance for LSF targets improved (lower tilt offset) at the expense of HSF detection (*b*). Finally, fear bradycardia was significantly correlated to the improved LSF detection (*c*). These findings indicate that freezing facilitates processing of coarse rather than detailed information (Adapted from Lojowska *et al*. [10])

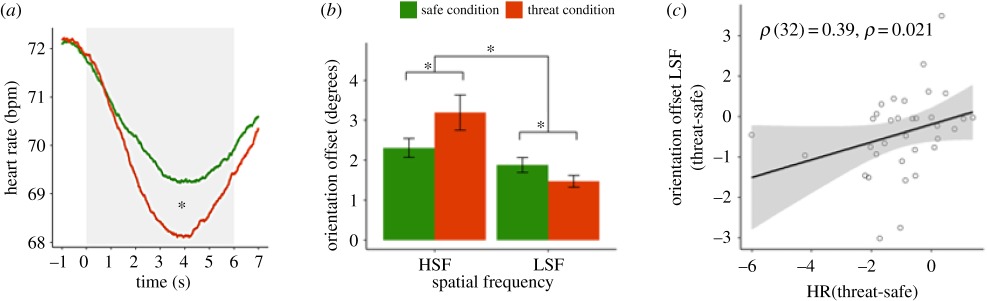
.

### (b) Freezing and action preparation

Second, to test the role of freezing in action preparation, Gladwin *et al*. [5] combined electrocardiographic and posturographic analyses in an action paradigm. The authors induced freezing (heart rate reduction) by threat of shock and found that heart rate reduction and body sway reduction were more pronounced in a condition where participants could actively prepare for action. In a shooting paradigm participants were presented with a target (one of two identities) that either signalled threat of shock (threat-condition) or safe condition. After a variable time interval, the target pulled a phone or a gun (cue), upon which the participant had to respond as fast as possible by shooting (go) or withholding (no-go), respectively. In one condition, the participant was armed and he or she could respond; however, in an unarmed condition, the participant could not prevent the shock by shooting (learned helplessness condition). Interestingly, the threat-induced freezing was stronger in the armed than in the unarmed condition. In addition, fear bradycardia increased when the subjects moved closer to the response time [5]. The role of freezing in action preparation has been further validated by a follow-up study showing that stronger PAG activity during freezing was associated with faster subsequent cue-signalled responding [81].

## 7. Neural correlates of freezing in humans

Neuroimaging studies in humans have indicated that, in humans, brain structures similar to those previously observed in animals (rodents and primates) are implicated in freezing. For example, Mobbs *et al*. [82–84] showed that activity in amygdala–PAG circuitry varies with threat proximity. Moreover, recent fMRI studies have suggested that activity in these circuits is associated specifically with freezing and its accompanying parasympathetic autonomic response [77]. Heart rate deceleration associated with aversive versus neutral picture-viewing in an MRI scanner was associated with increased activity in the PAG and increased connectivity between the amygdala and the PAG. On the basis of trial-by-trial correlations, the authors showed that the increased threat-induced PAG activity was specifically related to the parasympathetically driven heart rate reduction and did not occur as a function of sympathetic index (i.e. pupil dilation). Even when there was controlled for pupil dilation, the heart rate modulations of PAG activity remained statistically significant. Interestingly, the maximum of this partial correlation was located in the ventral part of the PAG. Although this finding should be interpreted with caution, given the limited spatial resolution of blood oxygen level-dependent (BOLD)–fMRI, it is consistent with a functional segregation of the PAG described in the animal literature [48]; figure 2). We have recently replicated these effects of increased PAG activity in relation to heart rate deceleration during threat of shock [81]. By combining threat of shock with active responses in the above-described shooting paradigm, we showed that the transition from PAG-mediated freezing to active fight was associated with activity in the perigenual ACC and the amygdala [81]. These findings provide preliminary evidence that humans, just like animals, recruit amygdala, PAG and ventral forebrain structures to control the defence cascade [62]. These structures also support learning of behavioural responses to pain. For example, Roy *et al*. [85] showed that the PAG was involved in encoding learning signals during pain avoidance learning and transferring those signals to frontal regions implicated in behavioural control, including perigenual ACC, dorsomedial PFC and orbitofrontal cortex [85].

## 8. Individual differences in human freezing

Stronger freezing reactions observed in anxious and traumatized rodents [27,70,86] have given rise to the exploration of individual differences in freezing as a function of anxiety and previous adverse events in humans. Indeed, we found that freezing reactions to angry faces (versus neutral and happy), quantified by reductions in body sway and heart rate, were correlated with self-reported levels of state anxiety [1]. State anxiety also affected body sway in a study comparing frequencies of the postural sway power spectrum in highly and lowly anxious participants [87]. Finally, Lopes *et al*. [88] found reduced body sway in response to several types of affective pictures as well as a general decrease in body sway throughout the experiment as a function of self-reported anticipatory anxiety in patients with panic disorder.

As far as associations between freezing and history of adverse events, Hagenaars *et al*. [89] found stronger freezing reactions to aversive IAPS pictures (compared with neutral and appetitive) in previously traumatized individuals. In a prospective longitudinal study, Niermann *et al*. [2] observed increased freezing in 14-year-old adolescents who were classified with insecure parent–child attachment in infancy (15 months of age). Compared with adolescents who were classified as being securely attached in infancy, they showed reduced body-sway reactions to angry (compared with happy and neutral) facial expressions. Together, these findings suggest that defensive reactions in humans may be sensitive to anxiety and previous adverse events. Just like in animals [70], the latter study suggests that early-life adversities may have long-lasting effects in humans. Further research is needed to investigate whether freezing may be a relevant biomarker for psychopathology. First hints in this direction are provided by a recent study in our laboratory, demonstrating that poor recovery of stress-induced freezing mediates the relation between blunted HPA axis activity and internalizing symptoms [90].

## 9. Outlook

Freezing is one of the main defensive threat reactions across species. Although defensive threat reactions in animals [8] are at the basis of human models of defensive responding [15,30,91], only recently researchers have started to explore the behavioural features of bodily freezing in humans. The promise of this route has become evident from various observations. First, apart from across-species differences, there are striking similarities in the core phenomenology and neural correlates underlying freezing in animals and humans, validating the use of cross-species models of defensive threat responses. Second, building on animal research, human studies have contributed insights into the role of freezing in action preparation. Freezing may be a special case of threat-induced motor inhibition. Conceptualization of freezing as an active action preparatory state with a parasympathetic amygdala–vlPAG-driven ‘brake’ on the system may help to understand rapid adaptive responding once the brake is ‘released’ by frontal–amygdala connections. Third, human research is particularly useful in exploring perceptual changes associated with the parasympathetic-dominated state of freezing and has offered novel insights into low versus high spatial frequency sensitivity during freezing, which are worth translating into animal research. Finally, building on animal models, research in human developmental and clinical samples has provided starting points for investigating the role of freezing in the development of psychopathology. Yet, there are still many unknowns and an agenda should be set for future research to further advance translational animal to human research and vice versa.

In animals and humans, the parasympathetic state of freezing has been associated with increased activation of and connectivity between the amygdala and the PAG. However, given the limited spatial resolution of BOLD–fMRI, there is need for more high-resolution imaging of the PAG to enable partialization of the PAG and investigate the role of human vlPAG and dlPAG in freezing and fight-or-flight, respectively [92]. In addition, this defensive fear network is part of a larger network encompassing ventromedial and ventrolateral PFC as well as ACC implicated in regulation of emotional behaviour and salience processing [73,93,94]. In two studies, we found that the perigenual ACC is particularly involved when people shift from freezing to active fight [81]. High-resolution imaging during active experimental paradigms may enable partialization of the PAG and its connections to establish the routes by which flexible shifting between defensive modes is organized [92].

A related unresolved issue deals with the question whether cortical motor areas are involved in threat-induced freezing reactions. It has been shown that emotional information can modulate the supplementary motor area's influence on primary motor cortex excitability during emotion-triggered movements [95]. Also, a recent transcranial magnetic stimulation study indicated that watching pictures of bodies expressing fear suppressed intracortical facilitation of the primary motor cortex, suggesting that the motor cortex may implement suppression of motor readiness when seeing emotional body expressions [96]. Hermans *et al*. [77] observed that threat-induced heart rate deceleration indicative of freezing was associated with activity in not only the amygdala and the PAG, but also in the supplementary motor cortex and the right inferior frontal gyrus. Future studies should explore whether and how motor cortex and frontal motor inhibitory areas interact with amygdala–PAG circuitries during freezing.

Another unexplored area is the neural pathways by which threat affects visual processing during freezing. Some studies have suggested that emotional cues have a selective effect on visual perception, enhancing perception of coarse visual features or LSF information, at the expense of fine-grained details, or high-spatial frequency (HSF) information [10,92,97]. Lojowska *et al*. [10] demonstrated that this effect was specifically associated with parasympathetic heart rate deceleration during freezing and not with sympathetic responses. LSF information is conveyed rapidly along the magnocellular pathway, which projects to the dorsal visual stream involved in action modulation [98,99]. It remains to be determined whether and how preferred LSF detection during freezing may be facilitated by this neural mechanism.

Revealing neurocognitive control of defensive threat reactions in humans is critical for people in high-risk professions who have to make split-second decisions. For example, police officers often have to decide whether or not to shoot on the basis of limited perceptual input. Optimal timing of defensive freezing and fight-or-flight reactions can be the essence in ensuring optimal visual input, action preparation and action decision. Current training programmes for snipers already focus on self-regulation of psychophysiological states, but this could also be relevant to all kinds of decisions under threat.

There are individual differences in the tendency to display freeze, fight or flight reactions to threat. Aggression has been linked to reduced freezing in passive situations [5] and increased freezing followed by fast fight decisions when possible in active threat-responding paradigms [3–5]. In contrast, anxiety has been linked to increased freezing and flight [1,87,88]. Moreover, prolonged presence of threat-induced freezing mediated the relation between blunted cortisol and internalizing symptoms [90]. Although these are first hints that defensive reactions may play a key role in emotion regulation and symptom development, there is a great lack of longitudinal studies investigating the development of defensive reactions and their roles in the development of psychopathology. On a theoretical note, it has been argued that automatic defensive reactions play an important role in emotion regulation [100,101] and in fact are the basis of emotional perception [101,102]. But the only studies in which freezing was actually linked to development of anxiety disorders such as post-traumatic stress disorder were based on retrospective self-reports of immobility during trauma [28,103,104]. It is important for future investigations to assess defensive response modes objectively, as they may form important risk markers as well as resilience markers for the development of psychopathology.

Finally, despite the wealth of studies on the effects of anxiolytic drugs on freezing in animals [41,73], there is a great lack of studies investigating those effects on automatic defensive reactions in humans. Particularly, serotonin-related drugs are an important target for future research because selective serotonin reuptake inhibitors are frequently applied in anxiety treatments (with mixed results) and because serotonin shows complex interactions with freezing, on the one hand, and active fight-or-flight reactions, on the other hand [73].

In conclusion, freezing is a form of behavioural inhibition accompanied by parasympathetically dominated heart rate deceleration. Despite the potential relevance of freezing for human stress-coping, its phenomenology and neurobiological underpinnings in humans remain largely unexplored. This review paper indicates that freezing is not a passive state but rather a parasympathetic brake on the otherwise active motor system, relevant to perception and appropriate action preparation. The currently presented model summarizes emerging evidence from animal and human investigations that detail the neural mechanisms involved in freezing and the switch to fight-or-flight action. Study of defensive responses and their neurobiological underpinnings along these lines may be relevant to advance insights into human stress resilience as well as stress vulnerability.
